# Structural characterization of laminarin and its preventive effects on the gut microbiota–butyrate–pancreas axis in severe acute pancreatitis

**DOI:** 10.1016/j.fochx.2026.104731

**Published:** 2026-10-30

**Authors:** Xinyu Deng, Zhaojie Xia, Kang Chen, Yawen Ye, Caixia Sun, Yiwei Yang, Ziwei Niu, Yaoyao Yang, Xiaohu Luo, Wei Liang, Lingling Jia

**Affiliations:** a College of Food Science and Engineering, Ningbo University, Ningbo, Zhejiang 315211, China; b Department of Laboratory Medicine, The First Affiliated Hospital of Ningbo University, Ningbo, Zhejiang 315010, China; c State Key Laboratory for Managing Biotic and Chemical Threats to the Quality and Safety of Agro-Products, Institute of Plant Virology, Ningbo University, Ningbo 315211, China; d Zhejiang Engineering Research Center of Innovative technologies and diagnostic and therapeutic equipment for urinary system diseases, Ningbo, Zhejiang 315210, China

**Keywords:** Laminarin, Fermentation, Gut microbiota, Butyrate, Severe acute pancreatitis, Functional food ingredient

## Abstract

Many dietary polysaccharides can modulate gut microbiota and promote beneficial metabolite production, yet the fermentation characteristics and physiological effects vary across different compounds. Laminarin, a β-(1 → 3)/(1 → 6)-glucan from the edible brown alga *Laminaria digitata*, has attracted interest as a functional food ingredient. In this study, we systematically characterized laminarin's structural features, evaluated its specific fermentability *via in vitro* fecal fermentation, and investigated its biological activity and preventive effects in a murine model of severe acute pancreatitis (SAP). Oral pretreatment with laminarin reshaped the gut microbiota, enriched short-chain fatty acid–producing genera, and significantly increased colonic butyrate levels. Elevated butyrate was associated with enhanced intestinal barrier integrity, reduced bacterial translocation and lipopolysaccharide accumulation, and attenuation of downstream inflammatory cascade, ultimately alleviating pancreatic injury. These findings reveal a microbiota-mediated mechanism link between laminarin fermentation, gut barrier reinforcement and pancreatic protection, highlighting laminarin as a fermentable substrate suitable for dietary prevention strategies in SAP.

## Highlights

• Structural features of laminarin and its SCFA kinetics during *in vitro* fermentation.
• Dietary laminarin reshapes gut microbiota and enhances colonic SCFA production.
• Laminarin pretreatment reinforces intestinal barrier integrity in SAP mice.
• Barrier reinforcement suppresses bacterial translocation and pancreatic inflammation.

## Graphical abstract

**Figure:**
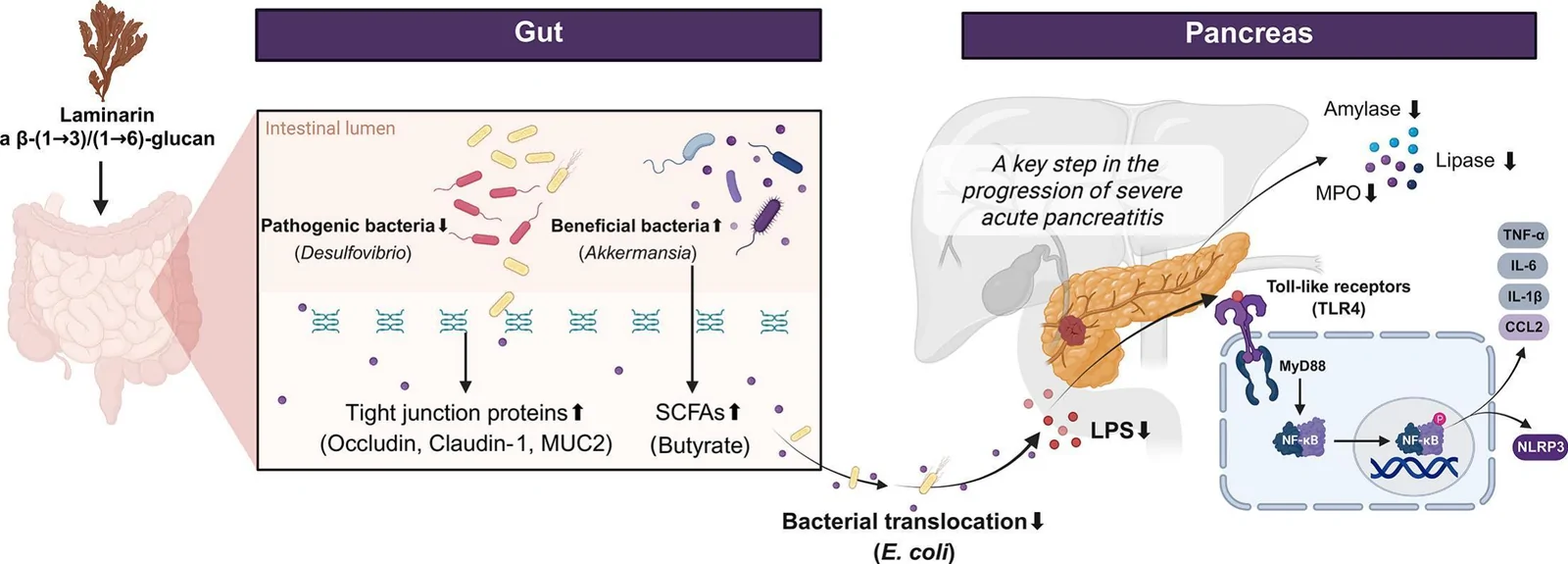
Proposed microbiota–gut–pancreas axis underlying the preventive effects of laminarin.

## 1 Introduction

Dietary polysaccharides are structurally diverse macromolecules whose biological activity can be influenced by molecular size, extraction methods, type of sugar, type of linkage and molecular geometry (Hussain & You, 2023). Among them, β-glucans have attracted considerable attention in food chemistry as potential prebiotics due to their capacity to modulate gut microbiota composition and promote short-chain fatty acid (SCFA) production (Chen, Guan, et al., 2022; Wu et al., 2025). SCFAs—particularly butyrate—play a pivotal role in maintaining intestinal epithelial integrity and regulating inflammatory responses (Li et al., 2024). Butyrate has been shown to reinforce tight junction assembly, enhance mucus secretion, and suppress pro-inflammatory signaling, thereby providing a mechanistic bridge between dietary polysaccharides, gut microbiota, and host homeostasis (Chen, Chen, & Fu, 2022; van den Berg et al., 2021). Consequently, dietary polysaccharides that promote butyrate-producing bacteria represent an important nutritional strategy for supporting intestinal health and mitigating inflammation-associated disorders (Wang et al., 2022).

Severe acute pancreatitis (SAP) is a life-threatening inflammatory disorder with high mortality, characterized by extensive pancreatic necrosis, systemic inflammatory response, and multiple organ failure (Petrov & Yadav, 2019). Increasing evidence indicates that intestinal dysfunction, particularly intestinal barrier disruption, is a pivotal driver of SAP progression (Hu et al., 2026). Impaired epithelial integrity facilitates bacterial translocation and lipopolysaccharide (LPS) leakage, triggering excessive inflammatory cascades that exacerbate pancreatic injury (Mei et al., 2021; Zhang et al., 2025). Although probiotics and prebiotics are widely recognized as major microbiota-based strategies to improve intestinal and host health (Sanders et al., 2019; Yan et al., 2023), clinically applicable agents that effectively and specifically target the gut–pancreas axis remain scarce.

Laminarin is a water-soluble β-(1 → 3)/(1 → 6)-glucan predominantly found in edible brown macroalgae such as *Laminaria digitata*. Owing to its structural resistance to host digestion, laminarin has attracted increasing attention in recent years for its notable prebiotic potential (Bishehsari et al., 2018; Guo et al., 2025; Shannon et al., 2021). It may contribute to host intestinal health by promoting gut microbiota homeostasis, enhancing intestinal barrier function, and mediating microbial metabolite-related benefits (Qianmin et al., 2025). However, although these promising properties make laminarin a suitable model compound, its preventive efficacy against SAP and the detailed mechanistic pathway linking its fermentation, butyrate production, intestinal barrier reinforcement, and pancreatic protection remain insufficiently elucidated.

In this study, we systematically characterized the structural features of laminarin extracted from *Laminaria digitata*, evaluated its SCFA production kinetics *via in vitro* fermentation, and further validated its downstream physiological effects using an *in vivo* model of SAP. Additionally, we aimed to investigate whether laminarin pretreatment could confer protection *via* a “gut microbiota–butyrate–pancreas” axis within the SAP context, examining its effects on gut microbiota remodeling, intestinal barrier reinforcement, and pancreatic injury mitigation. This work provides new insights into the potential application of laminarin as a functional food ingredient for dietary strategies aimed at preventing SAP.

## 2 Materials and methods

### 2.1 Materials

Caerulein was obtained from Jiangsu Jitai Biological Engineering Co., Ltd. (Jiangsu, China). LPS was sourced from General Laboratory Products (GLP, USA). The myeloperoxidase (MPO) assay kit was acquired from the Nanjing Jiancheng Bioengineering Institute (Nanjing, China). HiFiScript All-in-One RT Master Mix for qPCR and SuperStar Universal SYBR Master Mix were purchased from Jiangsu Cowin Biotech Co., Ltd. (Taizhou, China). ELISA kits for mouse TNF-α, IL-6, and IL-1β were obtained from Shanghai Weya Biotechnology Co., Ltd. (Shanghai, China). Primary antibodies against Claudin1, Occludin, MUC2, TLR4, MyD88, NF-κB p65, Phospho-NF-κB p65, and NLRP3 were obtained from ABclonal Technology (Wuhan, China), while the antibody against GAPDH was purchased from Abcam (Cambridge, UK). All other reagents used in this study were of analytical grade and commercially available.

### 2.2 Preparation of laminarin

Laminarin was extracted from dried *Laminaria digitata* according to the previous reports with minor modifications (Ashok et al., 2025; Rajauria et al., 2021). Briefly, the algal powder was defatted with 95% ethanol, then extracted with hot water in a ratio of 1:40 (*w*/*v*) at 90 °C for 2 h. After centrifugation, the supernatant was treated with CaCl_2_ to remove alginate and subsequently precipitated with three volumes of 95% ethanol. The precipitate was re-dissolved in hot water and incubated overnight at 4 °C to remove insoluble components. The solution was dialyzed (molecular weight cut-off 3.5 kDa) against distilled water. The crude polysaccharide extract was obtained from the dried algal powder at a yield of 7.84%. To separate neutral laminarin from sulfated fucoidan, the dialyzed extract was subjected to DEAE-Sepharose ion-exchange chromatography, eluted stepwise with water followed by increasing NaCl concentrations. The water-eluted neutral fractions were collected, desalted, and further purified by size-exclusion chromatography. The final laminarin fraction was recovered at 63.15% relative to the mass of the collected neutral fractions. The purified laminarin was lyophilized and characterized by FT-IR, monosaccharide composition analysis, and NMR spectroscopy before animal experiments.

### 2.3 Structural characterization of laminarin

#### 2.3.1 Determination of carbohydrate content

The phenol‑sulfuric acid method was employed to determine the carbohydrate content of laminarin (Dumez et al., 2006). Glucose standard solutions (0, 0.1, 0.2, 0.4, 0.6, 0.8, and 1 mg/mL) were used to construct the standard calibration curve. 0.1 mL of each standard solution and polysaccharides solution (1 mg/mL) was added to 0.1 mL of 6% phenol solution individually. After mixing thoroughly, 0.5 mL of concentrated sulfuric acid was added dropwise at once. The absorbance was measured at 490 nm using a TU-1901 double-beam UV–Vis spectrophotometer.

#### 2.3.2 Determination of ash content

Ash content was determined by dry ashing. Briefly, samples were mixed with magnesium acetate solution, carbonized, and subsequently ashed in a muffle furnace (SX-4-10, Shanghai Techeng, China) at 550 °C for 4 h until constant weight.

$$\mathrm{Ash}\;\text{content}\;\left(\%\right)=\frac{M_{3}-M_{1}}{M_{2}}\times 100$$

M_1_: crucible weight; M_2_: sample weight; M_3_: crucible plus residue weight.

#### 2.3.3 Determination of moisture content

Moisture content was determined by oven-drying at 105 °C for 3 h until constant weight.

$$\text{Moisture content}\;\left(\%\right)=\frac{W_{2}-W_{3}}{W_{2}-W_{1}}\times 100$$

W_1_: dish weight; W_2_: dish plus sample weight; W_3_: dish plus dried sample weight.

#### 2.3.4 Determination of protein content

Protein content was determined using a bicinchoninic acid (BCA) protein assay kit (Beyotime Biotechnology, Shanghai, China) according to the manufacturer's instructions, with bovine serum albumin (BSA) as the calibration standard. The reaction mixture was incubated at 37 °C for 30 min, and the absorbance was measured at 562 nm.

#### 2.3.5 Determination of sulfate content

Sulfate content was determined by the BaCl_2_–gelatin turbidimetric method. Briefly, 1 mg of the laminarin sample was hydrolyzed with 250 μL of 0.5 mol/L HCl at 100 °C for 3 h to release sulfate groups. The supernatant (20 μL) of the hydrolysate was mixed with 140 μL of 0.5 mol/L HCl, followed by the addition of BaCl₂–gelatin reagent (40 μL). After incubation at room temperature for 20 min, the absorbance was measured at 405 nm. Sodium sulfate (Na₂SO_4_) was used as the calibration standard.

#### 2.3.6 Total phenolic content (TPC)

Total phenolic content was determined by the Folin–Ciocalteu method. Samples (0.1 g) were extracted with 1 mL ethanol at 60 °C for 2 h, and the supernatant (10 μL) was reacted with Folin–Ciocalteu reagent (50 μL) and Na_2_CO_3_ solution (50 μL). The absorbance was measured using a multifunctional microplate reader (SpectraMax iD3, Molecular Devices, USA) at 765 nm with gallic acid as standard.

#### 2.3.7 Molecular weight (mw) distribution

Laminarin (5 mg) was dissolved in 0.1 mol/L NaNO_3_ (1 mL) for 1 h, filtered using a sterile syringe filter (0.22 μm PTFE membrane), and analyzed by GPC/SEC using an Agilent 1260 Infinity II system equipped with a refractive index (RI) detector and two PL-Aquagel-OH columns (7.5 × 50 mm, 8 μm; 7.5 × 300 mm, 8 μm). The mobile phase was 0.1 mol/L NaNO_3_. The system was operated at 45 °C and 1.0 mL/min, with an injection volume of 20 μL. Molecular weight calibration was performed using a series of polyethylene glycol/polyethylene oxide (PEG/PEO) standards with molecular weights ranging from 1470 to 454,000 g/mol. The calibration curve was constructed by plotting the logarithm of molecular weight (log Mw) against retention time, and the weight-average molecular weight of laminarin was determined from the resulting calibration curve. The PEG/PEO calibration curve is provided in Fig. S1.

#### 2.3.8 Determination of monosaccharide composition

Monosaccharide composition was determined by high-performance anion-exchange chromatography with pulsed amperometric detection (HPAEC-PAD). Briefly, 5 mg of the sample was hydrolyzed with 2 M trifluoroacetic acid (TFA) at 121 °C for 2 h. After hydrolysis, the solution was evaporated to dryness under a nitrogen stream, re-dissolved in deionized water, and filtered through a 0.22 μm membrane filter. Chromatographic analysis was performed on a Dionex ICS-5000+ system (Thermo Scientific) equipped with a CarboPac PA-20 anion-exchange column (3 × 150 mm, Dionex). Gradient elution was performed using a three-solvent system comprising water (A), 0.1 M NaOH (B), and 0.1 M NaOH/0.2 M sodium acetate (C) according to the gradient program detailed in Supplementary Table S1. The flow rate was maintained at 0.5 mL/min with an injection volume of 5 μL. Monosaccharide standards (L-fucose, L-rhamnose, L-arabinose, D-galactose, d-glucose, D-xylose, D-mannose, d-fructose, d-ribose, D-galacturonic acid, D-glucuronic acid, D-mannuronic acid, and D-guluronic acid) were used for calibration. Data acquisition and analysis were performed using Chromeleon 7.2 software (Thermo Scientific).

#### 2.3.9 Congo red assay

NaOH solutions at varying concentrations (0, 0.1, 0.2, 0.3, 0.4, 0.5, 0.6, 0.7, and 0.8 mol/L) were prepared. 2.5 mL Congo red (80 μmol/L) solution was mixed with 1 mL polysaccharide solution (2 mg/mL). Subsequently, 3 mL of each NaOH solution was added to the mixture to create alkaline conditions. Maximum absorption wavelength (λ_max_) was measured using a TU-1901 double beam UV–vis spectrophotometer in a 400–600 nm range after equilibrating for 1 h.

#### 2.3.10 Scanning electron microscopy (SEM)

The surface morphology of laminarin was examined using SEM (S-4800, Hitachi, Japan). Samples were mounted onto an aluminum disc and then coated with a thin layer of gold to enhance conductivity. The disc was subsequently placed in the SEM for imaging at magnifications of 20,000× and 50,000× to observe detailed surface features.

#### 2.3.11 Fourier-transform infrared spectroscopy (FT-IR)

FT-IR spectroscopy (Thermo Fisher Scientific, Nicolet iS20, Waltham, MA, USA) was employed to identify the functional groups present in laminarin. 2 mg of dried laminarin was accurately weighed, ground with 100 mg of potassium bromide (KBr), and pressed into a 1 mm pellet (Jin-Yang et al., 2025). The infrared spectrum was recorded over a wavenumber range of 4000 to 400 cm^−1^.

#### 2.3.12 Nuclear magnetic resonance spectroscopy (NMR)

1D (^1^H, ^13^C) and 2D (^1^H—^1^H COSY, HSQC) spectra were recorded using a Bruker Avance NEO 600 MHz spectrometer with a 5 mm prodigy QCI cryoprobe. 30 mg of laminarin was dissolved in 0.6 mL of DMSO‑*d*_6_ at 80 °C (Xia, Lin, et al., 2022). The sample was transferred to 5 mm NMR tubes for data acquisition. The acquired spectra were processed using the Mnova NMR software package (v14.0.0, MestreLab Research).

### 2.4 Fermentation assay of laminarin by fecal microorganisms

The *in vitro* fermentation of laminarin was conducted as reported previously with minor modification (Lang et al., 2025; Yang et al., 2025). The basal fermentation medium consisted of yeast extract (2 g/L), peptone (2 g/L), NaCl (0.1 g/L), KH_2_PO_4_ (0.04 g/L), K_2_HPO_4_ (0.04 g/L), MgSO_4_·7H_2_O (0.04 g/L), CaCl_2_ (0.01 g/L), NaHCO_3_ (2 g/L), hemoglobin (0.02 g/L), L-cysteine hydrochloride (0.5 g/L), bile salts (0.5 g/L), 0.2% Tween 80 (*v*/*v*), 1% resazurin solution (*v*/*v*), and vitamin K_1_ (10 μg/L). The pH of the fermentation medium was adjusted to 7.0 with 0.1 mol/L HCl solution and sterilized at 121 °C for 20 min. Fresh fecal samples were obtained from 18 male C57BL/6 J mice (6–8 weeks old, 18–22 g) housed at the Laboratory Animal Center of Ningbo University. The mice were maintained under specific pathogen-free (SPF) conditions with standard chow and autoclaved water *ad libitum*: room temperature of 23–25 °C, relative humidity of 40–60%, and a 12-h light/dark cycle. After a 7-day acclimation period, fecal samples were collected from mice that had received no experimental treatment. The animals were randomly divided into three independent donor groups (six mice per group). Fresh fecal samples from the six mice within each group were pooled to prepare one fecal inoculum, yielding three independent biological replicates (*n* = 3) for the *in vitro* fermentation experiment. For each biological replicate, the pooled fecal inoculum was distributed into four separate fermentation tubes corresponding to the four designated sampling time points (0, 6, 12, and 24 h). Each tube was incubated independently and was collected only once at its assigned time point; therefore, repeated aliquots were not taken from the same fermentation vessel. This approach minimized disturbance of the anaerobic fermentation environment during incubation. Fresh fecal samples were homogenized in PBS to obtain a 10% (*w*/*v*) suspension. *In vitro* fecal fermentation was performed in an anaerobic incubator (5% H_2_, 5% CO_2_, and 90% N_2_) at 37 °C. The fermentation system was prepared by mixing fermentation medium, fecal suspension, and test substrate solution at a ratio of 2:2:1 (*v*/*v*/*v*). Laminarin solution (20 mg/mL) served as the experimental substrate, while inulin solution (20 mg/mL) and sterile water were used as positive and negative controls, respectively. Samples were collected at 0, 6, 12, and 24 h and stored at −80 °C until SCFA analysis.

### 2.5 Animals and experimental design

The animals used in the experiment were male C57BL/6 J mice (6–8 weeks old, 18–22 g), housed at the Laboratory Animal Center of Ningbo University. All mice were acclimated for one week before the experiment. All animal experiments were approved by the Animal Ethics Committee of the Laboratory Animal Center of Ningbo University and were conducted in strict accordance with the National Research Council's *Guide for the Care and Use of Laboratory Animals* (License No. SYXK (Zhe) 2024–0002).

Mice were divided randomly into four groups (*n* = 12 per group): normal control (NC), model (SAP), SAP + low-dose laminarin (LAL, 100 mg/kg), and SAP + high-dose laminarin (LAH, 200 mg/kg). The dosages of laminarin were selected based on previous studies (He et al., 2023; Yun-Feng et al., 2022). Laminarin was administered intragastrically once a day for 14 consecutive days in the LAL and LAH groups; other groups were administered intragastrically sterile PBS. On day 15, mice in the SAP, LAL, and LAH groups were intraperitoneally injected with caerulein (50 μg/kg, 8 times at 1-h intervals), followed by lipopolysaccharide (10 mg/kg) immediately after the final caerulein injection, both dissolved in sterile normal saline (NS). Mice in the NC group received an equal volume of NS. Three hours after the last injection, mice were euthanized by a lethal dose of pentobarbital sodium (90 mg/kg; Sigma-Aldrich, USA). Samples (serum, colonic contents, pancreatic and colonic tissues) were subsequently collected and allocated to different downstream analyses. All 12 mice in each group completed the experiment, and no animals were excluded. For downstream analyses conducted with *n* = 6 per group, six mice were randomly selected from each group. The same six mice were used for histology, biochemical assays, gut microbiota analysis, SCFA measurement, and qPCR analysis of inflammatory cytokines and tight-junction-related genes. In particular, microbiota, SCFA, and qPCR data used for subsequent correlation analyses were obtained from the same individual mice to ensure matched measurements. Western blotting was performed as an independent confirmatory analysis of selected inflammatory and tight-junction proteins using three mice per group (*n* = 3).

### 2.6 Serum amylase and lipase assays

Serum samples were collected *via* retro-orbital bleeding, followed by centrifugation at 3000 ×*g* for 10 min at 4 °C. The serum amylase and lipase activities were measured using commercial kits (Mindray, Shenzhen, China) based on enzymatic kinetic principles, with a Beckman Coulter AU5800 automated biochemical analyzer according to the manufacturer's instructions.

### 2.7 Myeloperoxidase activity

Neutrophil infiltration in the pancreas was quantified by measuring tissue MPO activity according to the manufacturer's protocol. The absorbance was measured using a multifunctional microplate reader (SpectraMax iD3, Molecular Devices, USA) at 460 nm.

### 2.8 Histological analysis

Parts of the pancreas and colon tissue were fixed in 4% paraformaldehyde for 12 h, embedded in paraffin. After fixation, the tissue was cut into thin sections of 5 μm and then stained with hematoxylin and eosin (Ruchika et al., 2021). Histological scoring of the pancreas was performed using the modified Schmidt method, based on inflammatory infiltrate, acinar cell edema, vacuolization, and necrosis (Supplementary Table 2). And histological scoring of the colon was given based on inflammatory infiltrate, goblet cell loss, and crypt density (Supplementary Table 3). Histological scoring was performed with group allocation concealed from the investigators.

### 2.9 Immunohistochemical (IHC) assay

Colonic tissue sections were deparaffinized in xylene, rehydrated through a graded ethanol series, and subjected to antigen retrieval in 10 mmol/L sodium citrate buffer (pH 6.0) for 15 min. After blocking with 3% bovine serum albumin (BSA) at room temperature for 30 min, the sections were incubated overnight at 4 °C with primary antibody against Occludin. After washing, the sections were incubated with an HRP-conjugated secondary antibody (1:100) for 1 h. Immunostaining was visualized using a DAB horseradish peroxidase colour development kit (Beyotime Biotechnology, Shanghai, China). Images were captured using a DM3000 optical microscope (Leica, Germany) and quantified with ImageJ software.

### 2.10 Enzyme-linked immunosorbent assay (ELISA)

Serum LPS levels were quantitatively determined using a LPS (Lipopolysaccharide) ELISA kit (Elabscience, Wuhan, China), following the manufacturer's instructions. The absorbance was measured at 450 nm using a multifunctional microplate reader (SpectraMax iD3, Molecular Devices, USA).

### 2.11 RNA extraction and reverse transcription quantitative PCR (RT-qPCR)

Total RNA was isolated using the HiPure Total RNA Mini Kit (Magen Biotechnology Co., Ltd., Guangzhou, China) following the protocol. The concentration and quality of RNA were assessed using a NanoDrop ND-1000 spectrophotometer (Thermo Fisher Scientific, USA). RNA was reverse-transcribed by HiFiScript All-in-one RT Master Mix kit. The SuperStar Universal SYBR Master Mix kit was used to determine the mRNA levels of TNF-α, IL-1β, IL-6, CCL2, Claudin-1, Occludin, and MUC2. Relative expression of targeted genes was normalized to β-actin.Calculations were conducted based on the comparative cycle threshold method (2^-∆∆Ct^).

### 2.12 DNA extraction and quantitative PCR (qPCR) analysis

Genomic DNA was extracted from fecal samples using a fecal genomic DNA extraction kit (AJcore, China) and from pancreatic tissues using a tissue genomic DNA extraction kit (Servicebio, China). DNA concentrations were measured using a NanoDrop ND-1000 spectrophotometer. The relative abundances of *Escherichia coli*, *Faecalibaculum*, *Ruminococcus*, *Clostridium* cluster IV, and *Clostridium* cluster XIVa, as well as the relative abundances of the butyrate kinase (*buk*) and butyryl-CoA:acetate CoA-transferase (*but*) genes, were normalized to universal bacterial 16S rRNA gene abundance and calculated using the 2^-∆∆Ct^ method. The specificity of qPCR amplification was assessed by melting-curve analysis, with a single melting peak indicating specific amplification, and no amplification was detected in the no-template controls.

For absolute quantification of bacterial load in pancreatic tissue, DNA extracted from pancreatic samples was used as the qPCR template. Plasmid-based standard curves were generated using purified 16S rRNA gene fragments cloned into the pMD19-T vector. After plasmid extraction and linearization, copy numbers were calculated from plasmid concentration and molecular length, and 10-fold serial dilutions were prepared as standards. Absolute bacterial load was calculated from the standard curve and expressed as 16S rRNA gene copies per gram of pancreatic tissue. Melting curve analysis was performed to confirm amplification specificity.

The primers used in this study are provided in Supplementary Table 4.

### 2.13 Western blot analysis

Protein was isolated from colonic and pancreatic tissue lysates as per the previously reported method (Mudigunda et al., 2022). After the bicinchoninic acid (BCA) assay, protein samples were subjected to SDS-PAGE and electrotransferred to polyvinylidene difluoride (PVDF) membrane. The membrane was blocked with 5% nonfat milk and then incubated with primary antibodies against Claudin-1, Occludin, MUC2, TLR4, MyD88, NF-κB p65, phospho-NF-κB p65, and NLRP3, followed by incubation with the corresponding secondary antibodies. The multifunctional imaging system (ChemiDoc MP, Bio-Rad, USA) was used to develop the blots, and the protein band intensities were quantified using ImageJ software.

### 2.14 16S rRNA sequencing analysis

Total fecal microbial DNA was extracted by a Fecal Genome DNA Extraction Kit and amplified by PCR using universal primers 341F/805R (341F: 5’-CCT ACGGGNGGCWGCAG-3′; 805R: 5’-GACTACHVGGGTATCTAATCC-3′) (Logue et al., 2016). Qualifed PCR products were evaluated and further sequenced on an Illumina NovaSeq 6000 (PE250), provided by LC-Bio Technology Co., Ltd., Hangzhou, China. Raw sequences were processed using QIIME2. Briefly, sequences were denoised, quality-filtered, merged, and chimera-checked using DADA2 to generate amplicon sequence variants (ASVs). Taxonomic assignment was performed using the QIIME2 feature-classifier plugin against the SILVA database (Release 138). Feature abundances were normalized to relative abundances to construct the final ASV table.

Alpha (Chao1, Simpson and Shannon) and beta diversity were calculated using QIIME2. Taxonomy community was performed based on relative abundance. The Kruskal-Wallis test was employed to identify differentially abundant taxa at the phylum, family, and genus levels, and Benjamini–Hochberg (BH) correction was applied (FDR < 0.05), with the resulting adjusted values specifically denoted as *q* values. Linear discriminant analysis effect size (LEfSe; LDA ≥ 3.0, *p* < 0.05) was performed using nsegata-lefse to identify taxonomic biomarkers. Spearman correlation analysis was conducted to examine associations between differentially abundant genera and various physiological parameters, with *p* values adjusted for multiple testing using the BH procedure and the resulting FDR-adjusted values reported as *q* values. Correlations were visualized using hierarchical clustering heatmaps (Cheng et al., 2022). All other microbiota-related statistical analyses and data visualizations were performed using R software.

### 2.15 Quantification of short-chain fatty acids (SCFAs)

Seven target SCFAs, including acetic acid, propionic acid, butyric acid, isobutyric acid, valeric acid, isovaleric acid, and hexanoic acid, were quantified by gas chromatography–mass spectrometry (GC–MS). Briefly, sample was mixed with 30% phosphoric acid and acetone, homogenized for 3 min, and centrifuged at 12,000 ×*g* for 10 min. The supernatant was collected, and sample or standard solution was mixed with internal standard solution containing 2-ethylbutyric acid in 0.2 mol/L hydrochloric acid, followed by centrifugation. The resulting supernatant was directly injected into an Agilent 7820 gas chromatograph equipped with a DB-FFAP capillary column (30 m × 0.25 mm × 0.25 μm). The injection volume was 1 μL with a split ratio of 10:1, and high-purity helium was used as the carrier gas at a flow rate of 1.0 mL/min. The oven temperature was initially held at 70 °C for 5 min and then increased to 100 °C. Mass spectrometric detection was performed using an Agilent 5977 quadrupole mass spectrometer in selected ion monitoring mode. Data were acquired using MassHunter GC/MS Acquisition software.

### 2.16 Statistical analysis

All statistical analyses were performed using GraphPad Prism Software version 9.5 for Windows (GraphPad, SanDiego, USA). Continuous variables other than microbiota data are presented as the mean ± standard deviation (SD). Data normality was assessed using the Shapiro–Wilk test, and homogeneity of variance was evaluated using the Brown–Forsythe test. All continuous datasets analyzed under this framework satisfied the assumptions of normality and homogeneity of variance and were therefore analyzed using one-way ANOVA followed by Tukey's multiple-comparisons test. Histological scores, treated as ordinal data, were presented as the median with interquartile range (IQR) and analyzed using the Kruskal–Wallis test followed by Dunn's multiple-comparisons test. No data points were excluded as statistical outliers, and no missing data were present in the final datasets. Unless otherwise specified, *p* < 0.05 was considered statistically significant.

## 3 Results

### 3.1 Structural and compositional characterization of laminarin

Proximate composition analysis yielded the following values (%, *w*/w): total carbohydrate 81.33 ± 1.03, moisture 7.63 ± 0.73, ash 3.39 ± 0.24, protein content 1.57 ± 0.08, sulfate content 0.84 ± 0.04, and total phenolics 0.25 ± 0.01, which are consistent with previously reported laminarin preparations from brown algae (Xianglu et al., 2023). The quantified constituents accounted for the major composition of the preparation, while a minor fraction remained uncharacterized. Structural characterization further demonstrated its molecular identity. The results showed that laminarin is a homopolysaccharide composed exclusively of glucose (Fig. 1A), with a narrow molecular weight distribution (Mn = 11,089 g/mol, Mw = 12,272 g/mol, PDI = 1.107) indicating high structural uniformity (Fig. 1B). This structural simplicity has been associated with greater microbial accessibility and utilization of polysaccharides (Qian et al., 2020), raising the possibility that this feature may contribute to the fermentability of laminarin. The Congo red assay revealed a notable red shift (λ_max_ = 506 nm) in the laminarin–Congo red complex, consistent with an ordered triple-helix conformation (Fig. 1C). Such a secondary structure has been suggested in previous studies to contribute to the physicochemical stability and biological activities of polysaccharides (Ye et al., 2025). SEM imaging showed irregular, porous laminarin particles (Fig. S2), indicating an increased surface area that may enhance microbial accessibility (Han et al., 2020).

**Fig. 1:**
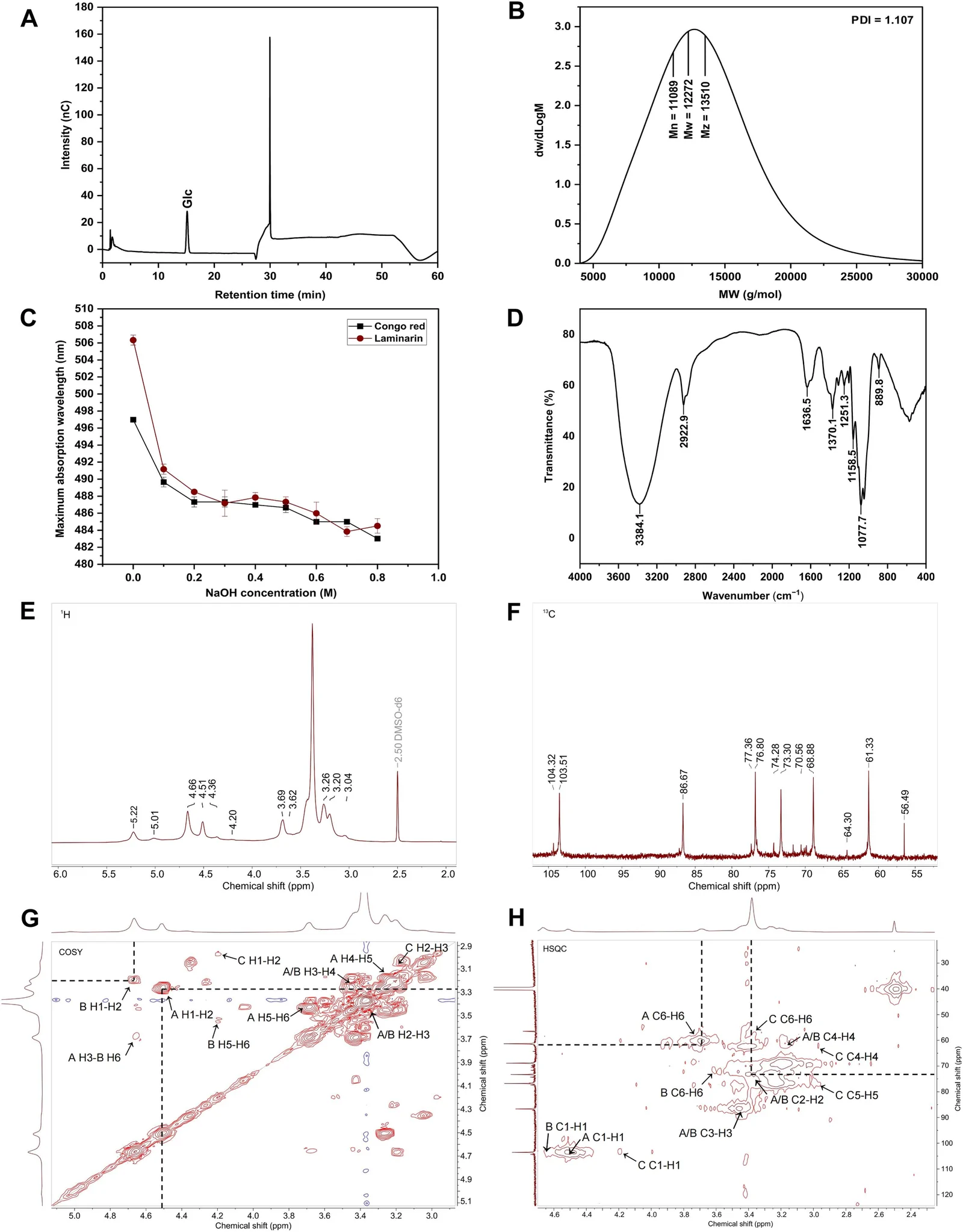
**Structural characterization of laminarin.** Monosaccharide composition (A), molecular weight distribution (B), Congo red conformational analysis (C), and functional group characterization of laminarin (D). Structural elucidation by ^1^H NMR (E), ^13^C NMR (F), ^1^H − ^1^H COSY (G), HSQC spectroscopy (H).

As shown in Fig. 1D, FT-IR analysis revealed abundant O − H groups at 3384 cm^−1^ (Liu, Gong, et al., 2018), C − H stretching at 2923 cm^−1^ (Yan et al., 2015), and C=O stretching at 1636 cm^−1^ (Pappas et al., 2004). The peaks at 1030–1070 cm^−1^ were attributed to C − O − C stretching vibrations, reflecting glycosidic linkages (Wang, Ma, et al., 2024). Importantly, the characteristic peak around 890 cm^−1^ confirmed the presence of β-glycosidic bonds, which distinguish laminarin from α-linked polysaccharides. This β-configuration has been proposed to generate beneficial metabolites that may be supportive of anti-inflammatory and barrier-protective effects (Yu, Zhu, et al., 2024). Further NMR spectroscopy was used to characterize the purity and structure of laminarin. In the ^1^H NMR spectrum (Fig. 1E), signals at *δ* 4.51 and 4.66 ppm were attributed to anomeric protons (H-1) of →3)-Glc*p*-(1 → (residue A) and →6)-Glc*p*-(1 → (residue B), respectively (Zhong et al., 2025). Additionally, the signal at *δ* 4.20 ppm indicated the presence of Glc*p*-(1 → (residues C). In the ^13^C NMR spectrum (Fig. 1F), signals at *δ* 104.32 and 103.51 ppm were assigned to C-1 of β-(1 → 3)- and β-(1 → 6)-linked glucoses (de Carvalho et al., 2025; Jiaqi et al., 2023). The C-3 carbon is involved in β-(1 → 3) linkages at *δ* 86.67 ppm, confirming the main chain structure. The 2D ^1^H—^1^H COSY spectrum (Fig. 1G) demonstrated proton–proton correlations (H1–H6), whereas the HSQC spectrum (Fig. 1H) exhibited proton–carbon correlations, further supporting the structural assignments (Gong et al., 2024). Collectively, NMR analysis confirmed the presence of β-(1 → 3)- and β-(1 → 6)-linked glucosyl residues in laminarin (Liu et al., 2014). These structural features are consistent with those of compounds possessing prebiotic properties, which may exert multifaceted regulatory effects on intestinal homeostasis and immunity.

### 3.2 Laminarin fermentation *in vitro* produced butyrate-enriched SCFA profile

As the principal fermentation products of dietary polysaccharides, SCFA concentrations are regarded as key indicators of intestinal microbiota activity (Wang et al., 2019). We performed *in vitro* fecal fermentation assays with laminarin as substrate and monitored SCFA production over 24 h to assess its fermentability. Individual SCFA analysis revealed time-dependent accumulation of acetic acid, propionic acid, butyric acid, and valeric acid (Fig. 2A–D). Notably, butyrate production was most pronounced during 12–24 h (*p* < 0.001) and comprised 28.84% of total SCFAs. This proportion is substantially higher than that typically generated by many common dietary fibers (Cui et al., 2021), suggesting that laminarin possesses a distinct capacity to selectively promote butyrate-producing bacteria. Inulin, a well-established prebiotic, was used as a positive control and significantly increased the total SCFA concentration at all time points as expected (Fig. 2E). After 24 h of fermentation, the total SCFA concentration in the laminarin group increased to 569.47 ± 61.39 μg/mL, which showed a marked increase relative to the blank group (185.80 ± 35.13 μg/mL) and comparable to that in the inulin group (731.49 ± 56.89 μg/mL), consistent with the reduction in pH observed in the laminarin and inulin fermentation cultures (Fig. 2F). Collectively, these *in vitro* data provide direct experimental validation that laminarin is a readily fermentable dietary fiber that drives a butyrate-enriched SCFA profile.

**Fig. 2:**
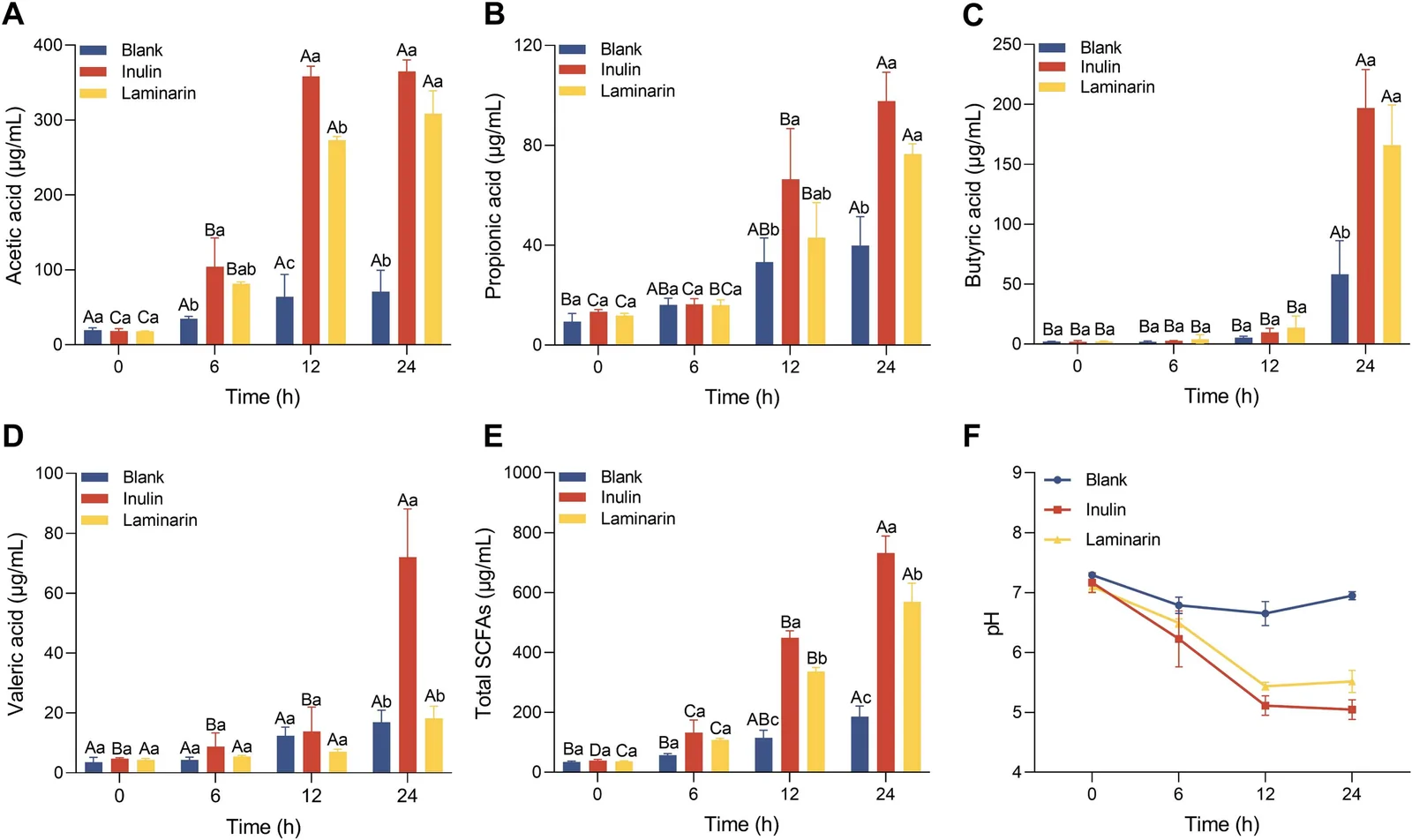
**Laminarin fermentation *in vitro* produced butyrate-enriched SCFA profile.** SCFA concentrations at different time points during *in vitro* fermentation: acetic acid (A), propionic acid (B), butyric acid (C), and valeric acid (D). Total SCFAs were calculated as the sum of acetic acid, propionic acid, butyric acid, and valeric acid (E). pH value at different time points during *in vitro* fermentation (F). Different lowercase letters indicate significant differences among Blank, Inulin, and Laminarin groups at the same time point, while different uppercase letters indicate significant differences among different time points within the same treatment group, based on Tukey's multiple comparisons test (*p* < 0.05). Data are presented as mean ± SD (*n* = 3).

### 3.3 Laminarin pretreatment reduced the pathological hallmarks of SAP in mice

Having established the structural features and fermentability of laminarin, we next investigated whether these properties could translate into host protection under SAP-associated pathological conditions using an *in vivo* murine model (Fig. 3A). The key biochemical markers of SAP, serum lipase and amylase, were measured at 3 h post-induction, a time point corresponding to the peak of enzyme activity as established in previous studies (Liu, Wen, et al., 2018). Laminarin pretreatment resulted in a dose-related reduction in serum lipase and amylase levels (Fig. 3B, C). Consistent with these findings, laminarin administration also potently attenuated the elevated myeloperoxidase (MPO) activity observed in pancreatic tissues of SAP mice (Fig. 3D). Furthermore, SAP mice exhibited significantly increased serum concentrations of pro-inflammatory cytokines, including TNF-α, IL-6, and IL-1β. These elevations were reversed in mice pretreated with both low-dose (LAL) and high-dose (LAH) laminarin (Fig. 3E–G). Collectively, these findings indicated that laminarin pretreatment effectively alleviated SAP severity, as evidenced by the reduction in pancreatic injury markers and systemic inflammation. These observations led us to further investigate the associated mechanisms.

**Fig. 3:**
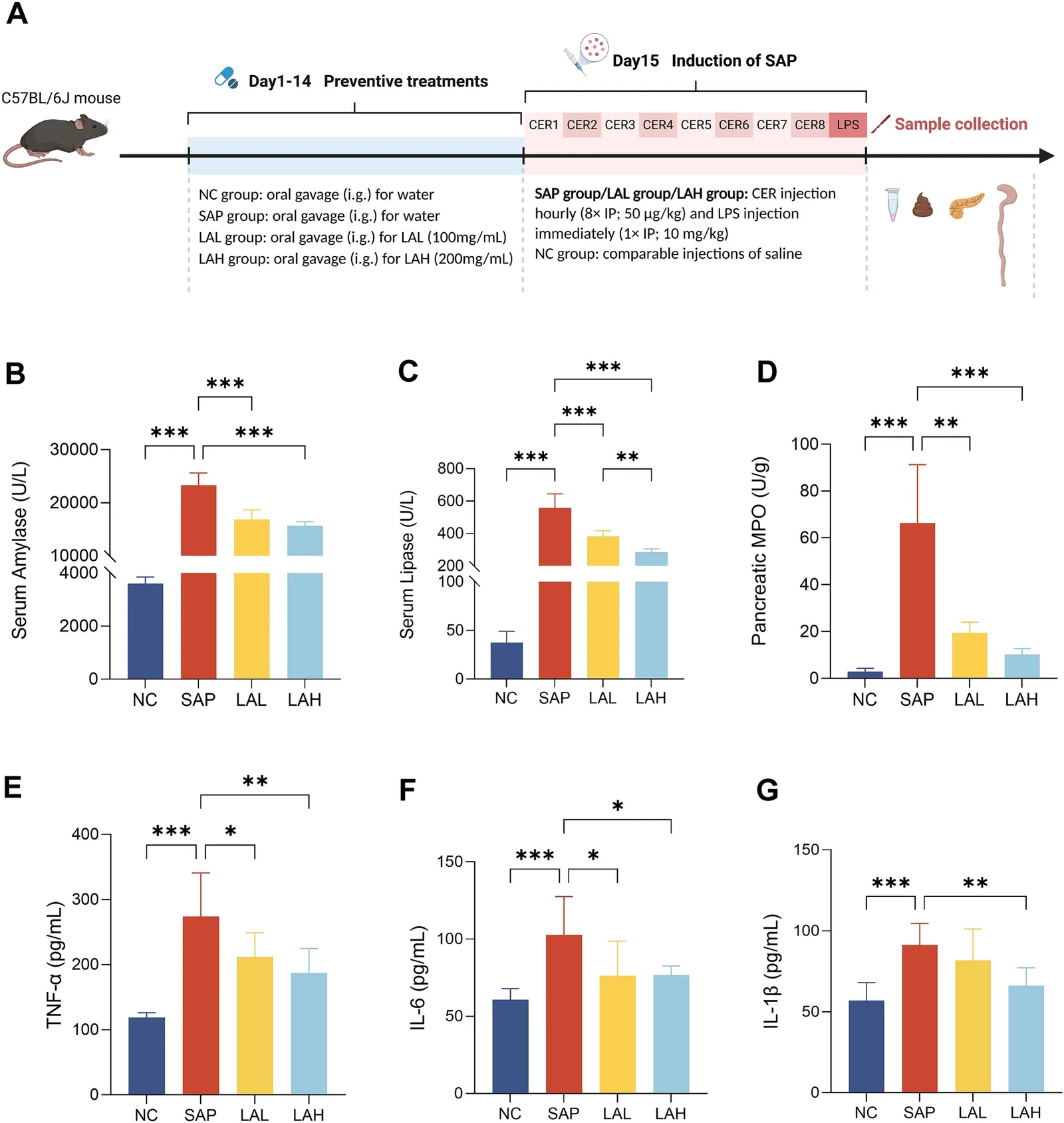
**Laminarin pretreatment reduced the pathological hallmarks of SAP in mice.** Mice were divided into normal control (NC) group, model (SAP) group, SAP + low-dose laminarin (LAL, 100 mg/kg) group, and SAP + high-dose laminarin (LAH, 200 mg/kg) group. (A) The diagram of the murine model of SAP establishment and pretreatment. Levels of (B) serum amylase, (C) serum lipase, and (D) pancreatic myeloperoxidase (MPO) activity. (E–G) ELISA analyses of TNF-α (E), IL-6 (F), and IL-1β (G) levels in serum. Data are presented as mean ± SD (*n* = 6/group). *p < 0.05, **p < 0.01, and ***p < 0.001.

### 3.4 Laminarin pretreatment alleviated pancreatic and colonic inflammation in SAP

During the development of SAP, TNF-α initiates inflammatory responses by inducing the production of various pro-inflammatory cytokines (IL-1, IL-6) and chemokines (CCL2) (Lin et al., 2025). These mediators, in turn, further upregulate TNF-α expression, thereby amplifying inflammation and aggravating tissue damage (Cuellar-Núñez et al., 2021). To evaluate the systemic anti-inflammatory effect of laminarin, we quantified the mRNA levels of these cytokines, along with the chemokine CCL2 in both pancreatic and colonic tissues. Laminarin pretreatment pronouncedly diminished the mRNA expression of these pro-inflammatory cytokines in both the pancreas (Fig. 4A) and colon (Fig. 4B), suggesting its systemic immunomodulatory capacity in attenuating SAP-induced inflammation. Histological analysis further corroborated the protective effects of laminarin, demonstrating reduced acinar edema, inflammatory cell infiltration, and vacuolization, along with better preservation of tissue architecture in the pancreas (Fig. 4C). Given the critical link between intestinal barrier dysfunction and SAP progression (Pan et al., 2023), we also examined colonic histology. Consistently, histological examination of colonic tissue revealed that laminarin considerably alleviated SAP-related intestinal injury, as evidenced by diminished inflammatory infiltration and crypt atrophy compared with the SAP group (Fig. 4D). Taken together, these findings suggested that laminarin ameliorated SAP-associated pancreatic and colonic injury, likely through modulating systemic inflammation and related immune signaling pathways.

**Fig. 4:**
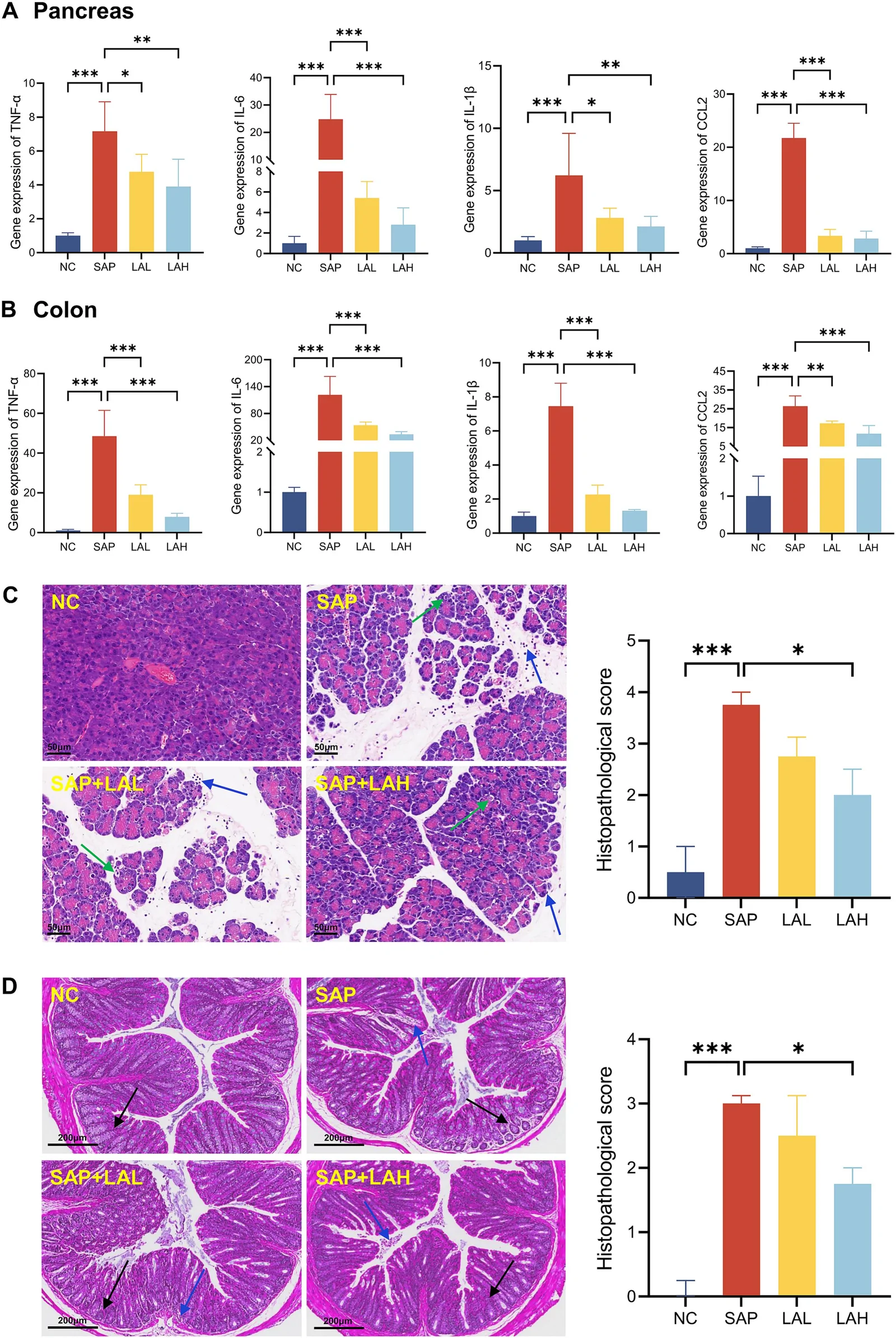
**Laminarin pretreatment alleviated pancreatic and colonic inflammation in SAP.** Mice were divided into normal control (NC) group, model (SAP) group, SAP + low-dose laminarin (LAL, 100 mg/kg) group, and SAP + high-dose laminarin (LAH, 200 mg/kg) group. (A, B) Relative mRNA expression of TNF-α, IL-6, IL-1β, and CCL2 in the pancreas and colon. (C) Representative H&E staining of pancreatic tissue and corresponding histopathological scores. The green arrows denote vacuolization, and the blue arrows denote inflammatory infiltration. Scale bar: 50 μm. (D) Representative H&E staining of colonic tissue and corresponding histopathological scores. Black arrows denote crypts and blue arrows denote inflammatory infiltration. Scale bar: 200 μm. Data in (A) and (B) are presented as mean ± SD, whereas histological scores in (C) and (D) are presented as median with interquartile range (IQR) (*n* = 6/group). Statistical significance was assessed using one-way ANOVA followed by Tukey's multiple-comparisons test for (A) and (B), and the Kruskal–Wallis test followed by Dunn's multiple-comparisons test for the histological scores in (C) and (D). *p < 0.05, **p < 0.01, ***p < 0.001. (For interpretation of the references to colour in this figure legend, the reader is referred to the web version of this article.)

### 3.5 Laminarin pretreatment suppressed pancreatic and colonic TLR4/NF-κB/NLRP3 signaling

The marked reduction in inflammatory cytokines prompted us to examine whether laminarin modulated key upstream signaling pathways. In SAP, compromised intestinal barrier integrity facilitates bacterial translocation and LPS release, which are known activators of the TLR4/NF-κB/NLRP3 signaling cascade (Mattke et al., 2024; Qi-Xiang et al., 2022). Accordingly, we evaluated pathway-related protein readouts in both tissues by Western blot (Fig. 5A, B). SAP significantly increased TLR4, MyD88, and NLRP3 protein levels and enhanced NF-κB p65 phosphorylation, whereas laminarin pretreatment effectively reversed these elevations. Collectively, these data indicated that laminarin attenuated pancreatic and colonic inflammation in association with the suppression of the TLR4/MyD88/NF-κB/NLRP3 signaling cascade. Given that TLR4 is the primary receptor for gut-derived LPS, the observed suppression of this pathway is consistent with the possibility that laminarin's protective effects may involve upstream actions to limit intestinal bacterial translocation. This prompted us to further investigate whether laminarin reinforced intestinal barrier integrity.

**Fig. 5:**
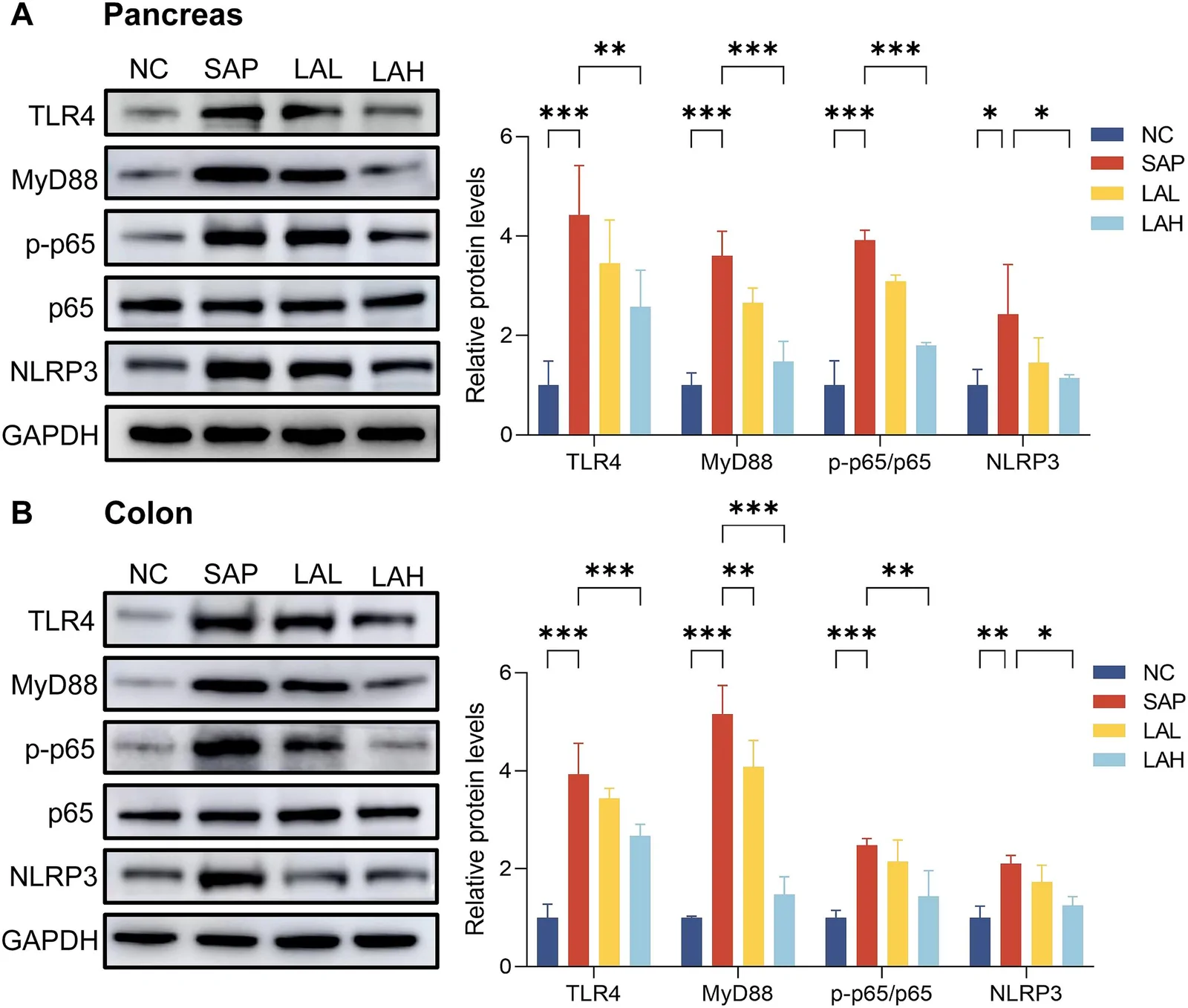
**Laminarin pretreatment suppressed pancreatic and colonic TLR4/NF-κB/NLRP3 signaling.** Mice were divided into normal control (NC) group, model (SAP) group, SAP + low-dose laminarin (LAL, 100 mg/kg) group, and SAP + high-dose laminarin (LAH, 200 mg/kg) group. Representative Western blot bands and quantitative analyses of TLR4/NLRP3 signaling pathway in pancreatic tissue (A) and colonic tissue (B). Data are presented as mean ± SD (*n* = 3/group). *p < 0.05, **p < 0.01, ***p < 0.001.

### 3.6 Laminarin pretreatment attenuated intestinal barrier disruption and bacterial translocation in SAP

Intestinal barrier disruption in SAP facilitates bacterial translocation and systemic LPS dissemination, which can activate pro-inflammatory signaling and thereby exacerbate disease progression (Xie et al., 2025). Therefore, we assessed intestinal barrier integrity and bacterial translocation as key readouts. Barrier function was evaluated by measuring the expression of tight junction proteins (Claudin-1 and Occludin) and the mucus barrier-associated protein MUC2. SAP markedly downregulated the expression of these barrier-related proteins compared with controls (*p* < 0.001). Laminarin pretreatment effectively restored their expression at both the transcriptional and protein levels (Fig. 6A–C, G, H), which is consistent with their essential roles in maintaining epithelial integrity (Cornick et al., 2019; Qijiang et al., 2023). Bacterial translocation was determined by quantifying serum LPS levels, total bacterial load (16S rRNA gene copies) and relative *E. coli* abundance in pancreatic tissue. Consistent with the restored barrier, laminarin administration significantly reduced the elevated serum LPS levels (Fig. 6D), decreased the total bacterial load in pancreatic tissue (Fig. 6E) and suppressed SAP-induced *E. coli* enrichment (Fig. 6F). Taken together, these data demonstrated that laminarin pretreatment reinforced intestinal barrier integrity and prevented bacterial translocation, thereby contributing to its dampening of TLR4-mediated inflammation. Given that intestinal barrier function is critically regulated by microbial metabolites, particularly SCFAs, we further investigated whether laminarin's barrier-stabilizing effects were associated with modulation of gut microbiota composition and SCFA production.

**Fig. 6:**
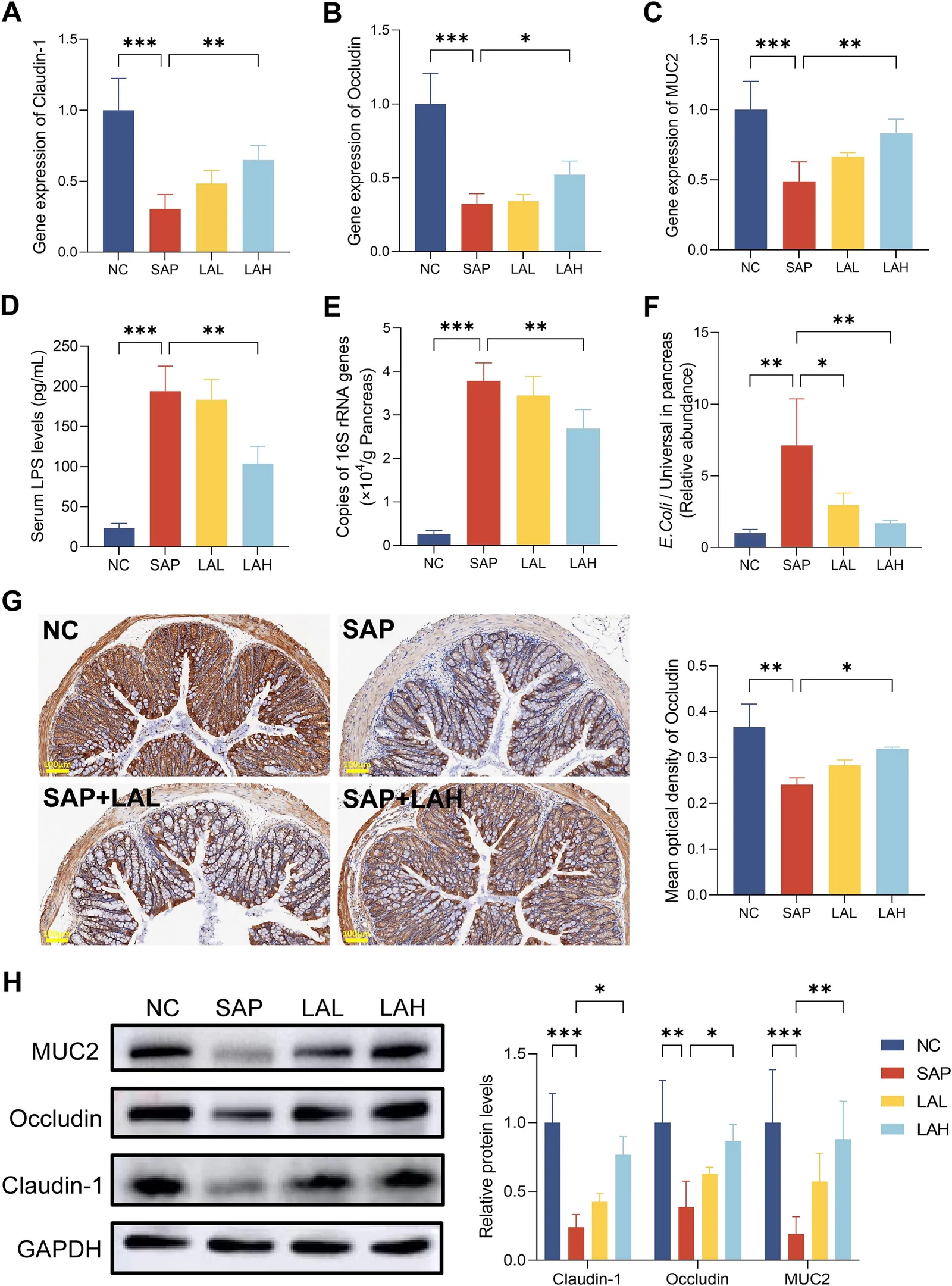
**Laminarin pretreatment attenuated intestinal barrier disruption and bacterial translocation in SAP.** Mice were divided into normal control (NC) group, model (SAP) group, SAP + low-dose laminarin (LAL, 100 mg/kg) group, and SAP + high-dose laminarin (LAH, 200 mg/kg) group. (A–C) Relative mRNA expression of tight junction proteins (Claudin-1, Occludin) and MUC2 expression in the colon (*n* = 6). (D) Serum lipopolysaccharide (LPS) concentrations (*n* = 6). (E) Total bacterial load expressed as 16S rRNA gene copies in the pancreatic tissues (*n* = 6). (F) Relative abundance of *Escherichia coli* (*E. coli*) in the pancreatic tissues (*n* = 6). (G) IHC analysis and quantification of Occludin in the colonic tissues (*n* = 6). Scale bar: 100 μm. (H) Representative Western blot bands and quantitative analyses of colonic Claudin-1, Occludin, and MUC2 (*n* = 3). Data are presented as mean ± SD. *p < 0.05, **p < 0.01, ***p < 0.001.

### 3.7 Laminarin pretreatment attenuated SAP-associated gut microbiota dysbiosis

The structure and composition of the gut microbiota in laminarin-pretreated mice were analyzed using 16S rRNA gene sequencing. The Venn diagram showed that the number of specific ASVs was 1390, 475, 682, and 703 in the NC, SAP, LAL, and LAH groups, respectively (Fig. 7A). Consistent with established SAP-associated dysbiosis, alpha diversity, encompassing species richness (Chao1 index) and community evenness (Simpson and Shannon indices) (Vigors et al., 2020; Wang, Li, et al., 2024), was significantly reduced in SAP mice relative to controls. High-dose laminarin supplementation partially restored these diversity indices (Fig. 7B). Principal coordinates analysis (PCoA) further revealed a clear separation in microbial community structure between the NC and SAP groups (Fig. 7C), while the laminarin-pretreated groups clustered more closely with the NC group, indicating a potential restorative effect on SAP-induced dysbiosis. Additionally, we analyzed gut microbiota alterations at the phylum (Fig. 7D) and family (Fig. 7E) levels. The relative abundance of beneficial phylum Firmicutes, which is associated with SCFA production, was decreased in SAP mice, whereas the pro-inflammatory phylum Proteobacteria was increased (Fig. S3A, B). These dysbiotic changes were partially reversed by laminarin. At the family level, laminarin restored the abundance of beneficial families, including Akkermansiaceae, Lachnospiraceae, and Erysipelotrichaceae, which are involved in SCFA production and intestinal homeostasis, whereas the abundance of pro-inflammatory Desulfovibrionaceae was potently depleted (Fig. S3C—F). This selective remodeling of the gut microbiota toward a health-promoting microbial community is essential for maintaining intestinal integrity. Most importantly, at the genus level, laminarin induced a selective remodeling of the microbiota. It significantly enriched beneficial genera, including *Akkermansia* (a mucin-degrading bacterium known to enhance barrier function) and key SCFA-producing genera such as *Lachnospiraceae_NK4A136_group*, *Ruminococcus*, and *Faecalibaculum* (Fig. 7F). qPCR analysis further supported these findings and revealed additional enrichment of butyrate-producing genera, specifically *Clostridium* cluster IV and *Clostridium* cluster XIVa (Fig. S4A—D). Conversely, laminarin suppressed multiple opportunistic and pro-inflammatory genera, including *Coriobacteriaceae_UCG-002*, *Desulfovibrio*, *Enterorhabdus* and *Tyzzerella* (Fig. 7G; Fig. S3G, H), thereby alleviating intestinal inflammation. Furthermore, the linear discriminant analysis (LDA) effect size (LEfSe) was performed to identify characteristic bacterial taxa among groups (Fig. 7H). Firmicutes dominated at the phylum level in the NC group, while SCFA-producing families, including Ruminococcaceae, Lachnospiraceae, and Clostridiaceae, were evidently enriched (LDA score > 3.0). Spearman correlation analysis (Fig. S5A—D) revealed that SCFA-producing genera among the 20 most abundant taxa, particularly *Lachnospiraceae_NK4A136_group* and *[Eubacterium]_ruminantium_group*, were inversely associated with systemic inflammatory cytokines, while exhibiting positive correlations with tight junction proteins and mucin-related markers. Conversely, opportunistic pro-inflammatory genera exhibited the opposite correlation pattern, suggesting a potential link between laminarin-driven microbial restoration and the amelioration of host inflammation and barrier dysfunction in SAP.

**Fig. 7:**
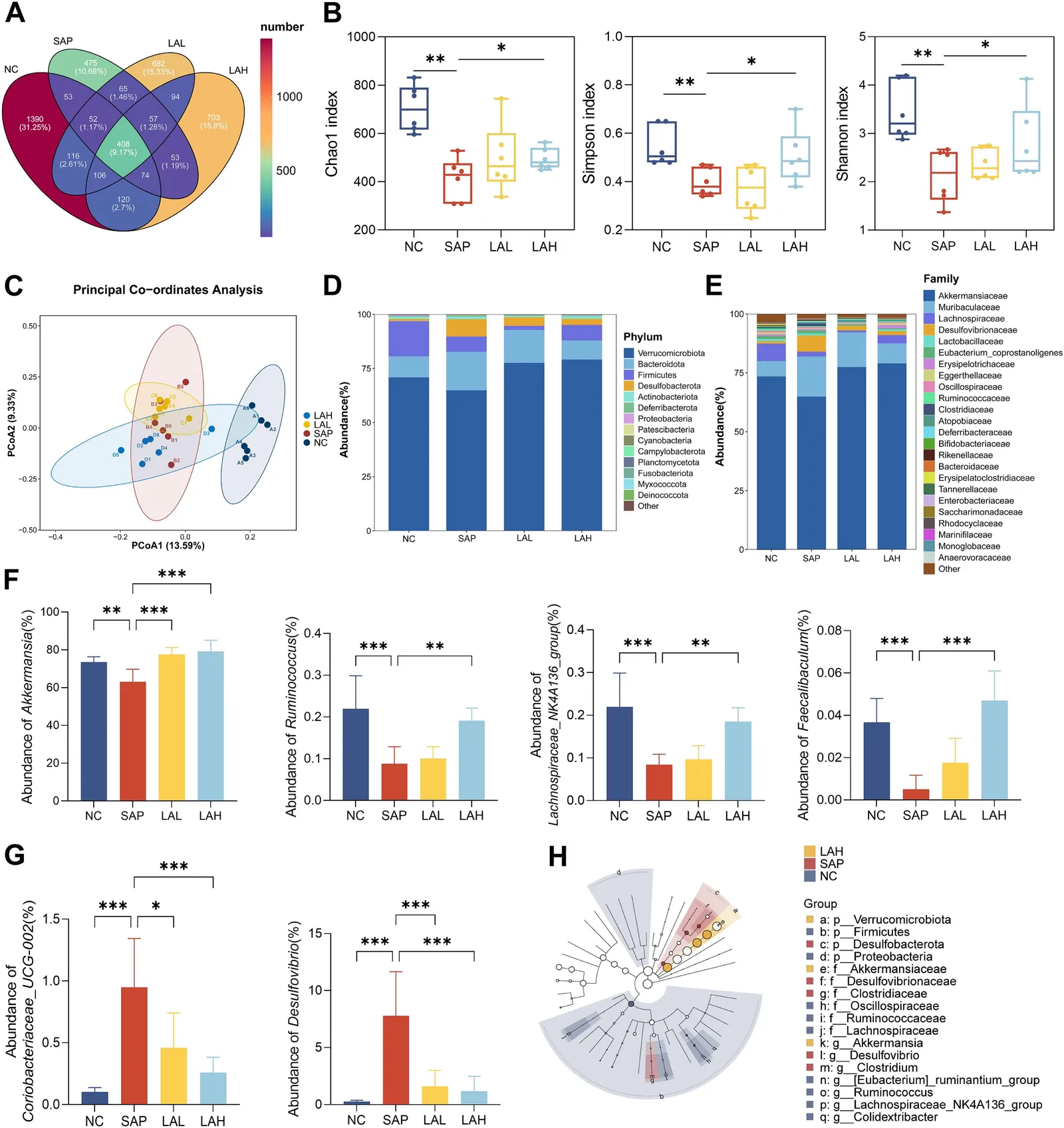
**Laminarin pretreatment modulated the gut microbiota composition in SAP mice.** Mice were divided into normal control (NC) group, model (SAP) group, SAP + low-dose laminarin (LAL, 100 mg/kg) group, and SAP + high-dose laminarin (LAH, 200 mg/kg) group. (A) Venn diagram on ASV level. (B) α-diversity indices (Chao1, Simpson and Shannon) among the four groups. (C) Principal coordinates analysis (PCoA) based on ASV level. (D, E) Alterations in gut microbiota composition among the groups at the phylum and family levels. (F) Relative abundance of *Akkermansia*, *Ruminococcus*, *Lachnospiraceae_NK4A136_group*, and *Faecalibaculum* among the groups. (G) Relative abundance of *Coriobacteriaceae_UCG-002* and *Desulfovibrio* among the groups. (H) Differentially abundant taxa identified by LEfSe analysis (LDA score > 3). Differential abundance analyses in (F) and (G) were performed using the Kruskal–Wallis test, with *p* values adjusted for multiple testing using the Benjamini–Hochberg procedure. FDR-adjusted *p* values are reported as *q* values. *n* = 6/group. *q < 0.05, **q < 0.01, ***q < 0.001.

### 3.8 Laminarin pretreatment restored SCFA production in SAP mice

As key microbial metabolites implicated in intestinal barrier integrity and immune regulation (Li et al., 2022), SCFAs in colonic contents were quantified to assess the metabolic outcomes of laminarin intervention. Consistent with microbiota dysbiosis, the concentrations of all seven detected SCFAs were evidently reduced in SAP mice (Fig. 8A–D; Fig. S6A—C). Notably, laminarin administration restored these metabolites, with a pronounced recovery of butyrate particularly in the LAH group (*p* < 0.001). To explore the relationship between microbial remodeling and metabolite output, we performed Spearman correlation analysis. The results revealed strong positive associations between SCFA concentrations and several butyrate-producing genera enriched by laminarin, including *Lachnospiraceae_NK4A136_group*, *[Eubacterium]_ruminantium_group*, and *Ruminococcus* (Fig. 8E). Furthermore, to further characterize the microbial potential for butyrate production, we quantified the abundance of two key genes involved in terminal butyrate biosynthetic pathways, the butyrate kinase (*buk*) gene and the butyryl-CoA:acetate CoA-transferase (*but*) gene (Gharechahi et al., 2021). High-dose laminarin pretreatment significantly increased the abundance of both *buk* and *but* genes (Fig. 8F, G), suggesting an increased genetic potential of the gut microbiota for butyrate production. These findings were consistent with the observed increase in butyrate levels and the enrichment of putative butyrate-producing bacteria.

**Fig. 8:**
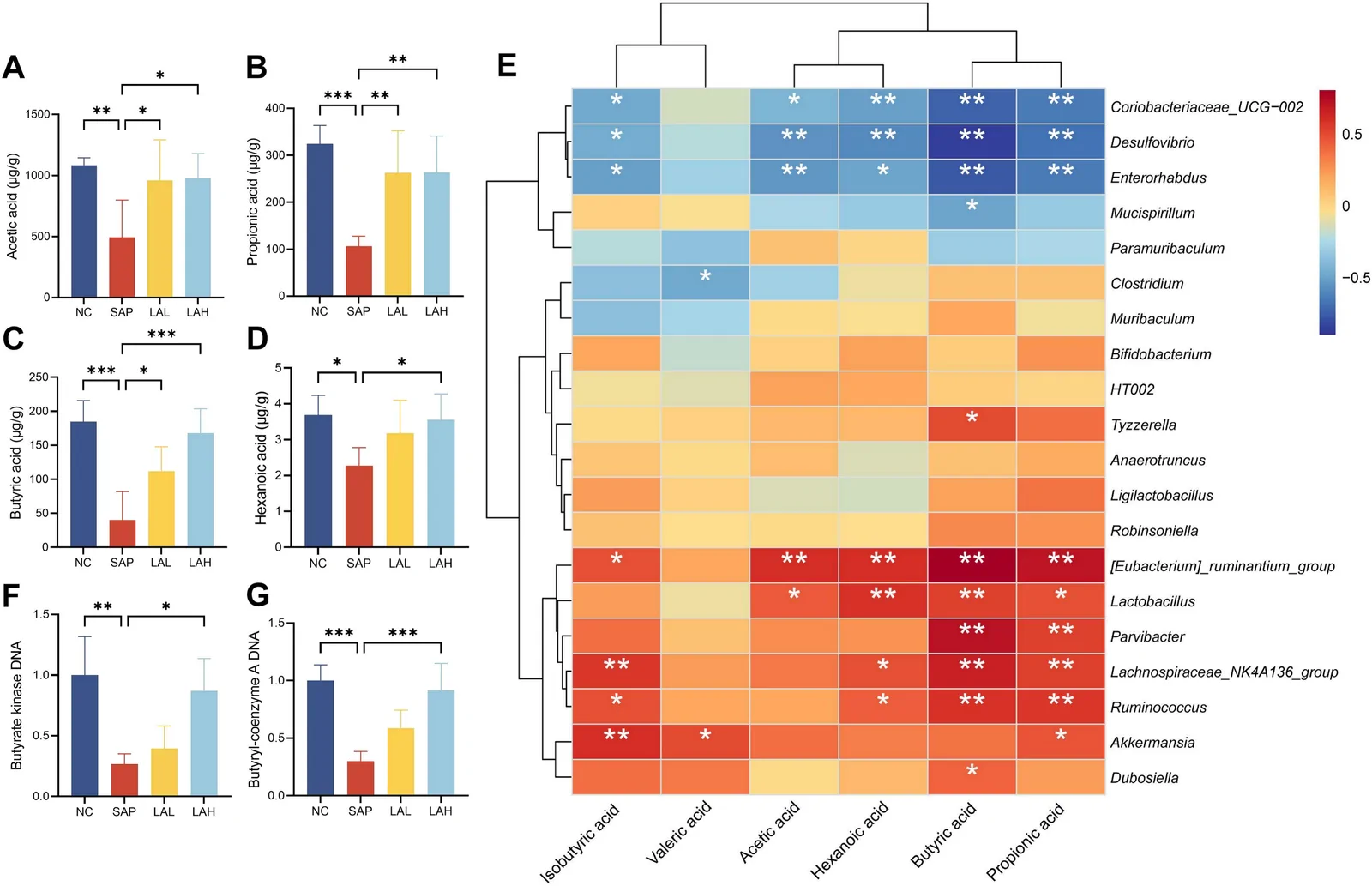
**Laminarin pretreatment restored SCFA production in SAP mice.** Mice were divided into normal control (NC) group, model (SAP) group, SAP + low-dose laminarin (LAL, 100 mg/kg) group, and SAP + high-dose laminarin (LAH, 200 mg/kg) group. Concentrations of SCFAs were quantified by GC–MS: (A) acetic acid, (B) propionic acid, (C) butyric acid, and (D) hexanoic acid. (E) Spearman correlation between the top 20 most abundant bacterial genera and SCFAs. (F, G) Relative gene abundance of *buk* (butyrate kinase) and *but-CoA* (butyryl-CoA). Data in (A–D) and (F, G) are presented as mean ± SD (*n* = 6/group) and were analyzed using one-way ANOVA followed by Tukey's multiple-comparisons test. Spearman correlation analyses in (E) were corrected for multiple testing using the Benjamini–Hochberg procedure, and FDR-adjusted *p* values are reported as *q* values. For (A–D) and (F, G), *p < 0.05, **p < 0.01, and ***p < 0.001; for (E), *q < 0.05, **q < 0.01, and ***q < 0.001.

## 4 Discussion

This study first systematically integrated structural characterization of laminarin with *in vitro* fermentation-based SCFA production kinetics, providing direct evidence for its prebiotic potential, and further demonstrated its downstream physiological effects using an *in vivo* model of SAP *via* a “gut microbiota–butyrate–pancreas” axis. With its β-(1 → 3)/(1 → 6)-glucan structure, laminarin functioned as a fermentable polysaccharide that reshaped the intestinal microbial community by enriching beneficial taxa (*e.g.*, *Akkermansia*) while suppressing potentially pathogenic bacteria (*e.g.*, *Desulfovibrio*). This microbial remodeling was associated with a marked increase in colonic butyrate levels and a consequential enhancement of intestinal barrier integrity, as evidenced by upregulation of tight junction (Claudin-1, Occludin) and MUC2. Improved barrier integrity was functionally linked to reduced bacterial translocation (*e.g.*, *E. coli*) and gut-derived LPS leakage, which in turn contributed to the dampening of the pancreatic and colonic TLR4/MyD88-dependent NF-κB activation and NLRP3 inflammasome cascade. Consistent with this plausible framework, laminarin pretreatment was associated with alleviated pancreatic injury and lower levels of systemic inflammatory mediators (TNF-α, IL-6, IL-1β, and CCL2). Collectively, our findings position laminarin as a microbiota-targeted, marine-derived agent with the potential to attenuate SAP progression through a coordinated gut–pancreas axis mechanism.

Dietary polysaccharides have attracted increasing interest in food chemistry because their molecular architecture critically affects their fermentability, microbial utilization, and downstream biological functions. Proximate composition and structural analyses showed that the laminarin used in this study is a β-(1 → 3)/(1 → 6)-glucan, whose β-glycosidic linkages are considered important structural features related to β-glucan bioactivity (Fig. 1B, D–H). Previous studies suggest that, unlike readily digestible α-linked polysaccharides such as starch, β-glucans can resist host digestion and reach the colon, where they may serve as fermentable substrates for specific bacterial populations (Jayachandran et al., 2018; Yu, Gao, et al., 2024). It is plausible that laminarin may potentially be available for microbial fermentation in the colon. In addition to linkage patterns, molecular weight has been reported to affect the accessibility and microbial utilization of polysaccharides (Pan et al., 2026). Accordingly, the moderately high molecular weight of the laminarin used in this study (Mw = 12,272 g/mol) might contribute to a more balanced and prolonged SCFA production profile (Chengxiao et al., 2021; Pan et al., 2022). The 24 h *in vitro* fermentation assay showed a time-dependent increase in SCFA production (particularly butyrate), providing direct evidence for the fermentability and prebiotic activity of laminarin (Fig. 2). This fermentation profile was further supported by the *in vivo* findings, in which laminarin pretreatment enriched SCFA-producing genera in SAP mice (Fig. 7F) and increased colonic SCFA levels (Fig. 8A–D). Furthermore, laminarin adopts a typical triple-helix conformation (Fig. 1C), a secondary structural feature widely implicated in the biological activities of polysaccharides, including immunomodulation relevant to intestinal homeostasis (Dong et al., 2025). Collectively, our structural characterization and fermentation results identify the physicochemical features of the laminarin preparation used here and demonstrate its fermentability. Together with the observed modulation of SCFA-producing bacteria and colonic SCFA levels *in vivo*, these results support the potential of laminarin for gut microbiota–SCFA modulation in SAP-related inflammatory injury.

The gut microbiota plays a dual role in AP, directly contributing to its onset and indirectly triggering associated inflammation, a factor correlated with disease severity (Zhou et al., 2024). Our investigation, consistent with earlier research indicating reduced microbiome diversity in SAP, revealed significant alterations in the diversity and composition of intestinal flora in SAP mice (Deng et al., 2024; Liu et al., 2023). Importantly, laminarin pretreatment markedly reversed SAP-associated gut dysbiosis, restoring microbial richness, diversity, and overall community structure toward a healthy state (Fig. 7A–E). Furthermore, laminarin selectively remodeled the gut microbiota toward a health-promoting configuration characterized by enrichment of SCFA-producing taxa (including *Akkermansia*, *Ruminococcus*, and *Lachnospiraceae_NK4A136_group*) and concomitant depletion of opportunistic and pro-inflammatory bacteria (Fig. 7F, G). Among these, *Akkermansia* can effectively stimulate mucin secretion to reinforce the intestinal mucus barrier (Zuo et al., 2022). Its documented barrier-protective properties, combined with the reported anti-inflammatory effects in AP models (Wen et al., 2024), suggest that *Akkermansia* enrichment may partially account for the therapeutic efficacy of laminarin in SAP management through gut microbiota modulation. Correlation analysis between the top 20 most abundant genera and systemic inflammatory markers, as well as colonic tight junction proteins, further suggested a potential link between microbial remodeling and host phenotypic changes (Fig. S5A—D). These microbiota–host associations may partly explain the systemic anti-inflammatory and barrier-protective effects (Fig. 3E–G; Fig. 6) observed in laminarin-pretreated SAP mice.

Laminarin-driven enrichment of SCFA-producing bacteria was accompanied by a marked increase in colonic SCFA levels, particularly butyrate, together with the upregulation of key butyrate biosynthesis genes (*buk* and *but-CoA*) in laminarin-treated mice (Fig. 8A–D, F–G). As the primary energy substrate for colonocytes and a known regulator of barrier-related gene transcription, butyrate maintains epithelial metabolic homeostasis and promotes tight junction assembly (*e.g.*, Claudin-1, Occludin) (Ara et al., 2016). Additionally, consistent with previous reports on epigenetic regulation by butyrate, this SCFA epigenetically upregulates MUC2 expression, thereby further fortifying the mucus barrier (Xia, Zhong, et al., 2022). Consistent with these established butyrate-mediated mechanisms of barrier fortification, laminarin-pretreated mice exhibited substantially upregulated expression of these barrier-related proteins (Fig. 6A–C, G, H), indicating that the elevated butyrate can functionally translate into enhanced intestinal barrier integrity. This barrier-protective effect was further supported by reduced total bacterial load and decreased *E. coli* translocation in the pancreas (Fig. 6E, F), thereby disrupting the initiation and amplification of pancreatic inflammation.

The pathophysiological importance of reinforcing the intestinal barrier lies in the gut–pancreas vicious cycle underlying SAP. Pancreatitis-associated proinflammatory cytokines compromise intestinal mucosal integrity, facilitating bacterial translocation and LPS leakage, which in turn exacerbate pancreatic injury and perpetuate barrier dysfunction (Sun et al., 2024; Wang et al., 2012). Central to this cycle is the TLR4/NLRP3 signaling, which may represent a molecular link between compromised intestinal barrier integrity and pancreatic injury. Our data demonstrate robust activation of the TLR4/NF-κB/NLRP3 pathway in both pancreatic and colonic tissues during experimental SAP (Fig. 5), paralleling heightened pro-inflammatory cytokine production (Fig. 3E–G; Fig. 4B, D). Critically, laminarin intervention disrupted this gut–pancreas inflammatory loop by fortifying intestinal barriers, effectively curtailing LPS-driven bacterial translocation and downstream inflammatory cascade.

In conclusion, the present study suggests that the fermentability and prebiotic potential of laminarin can contribute to intestinal health and mitigate pancreatic injury in the context of SAP, thereby extending its potential application as a marine-derived functional ingredient in preventive dietary intervention against SAP. Nevertheless, causal validation of the microbiota contribution, such as fecal microbiota transplantation or antibiotic depletion experiments, remains necessary. Although two doses of laminarin were included in the present design, a more comprehensive dose–response analysis with additional dose levels is warranted to define the optimal effective range for future functional food applications. Beyond its microbial-mediated effects, the possibility of an additional, direct structure-dependent interaction between laminarin and host receptors (*e.g.*, TLR4) merits further biophysical and cellular investigation to fully elucidate its mechanism of action. Collectively, these insights support laminarin as a promising functional β-glucan for butyrate-oriented nutritional modulation of inflammation-associated disorders.

## CRediT authorship contribution statement

**Xinyu Deng:** Writing – review & editing, Writing – original draft, Methodology, Investigation, Formal analysis, Data curation. **Zhaojie Xia:** Methodology, Investigation, Formal analysis, Data curation. **Kang Chen:** Writing – review & editing, Methodology, Formal analysis. **Yawen Ye:** Validation, Methodology, Conceptualization. **Caixia Sun:** Writing – review & editing, Methodology. **Yiwei Yang:** Methodology, Investigation. **Ziwei Niu:** Methodology, Investigation. **Yaoyao Yang:** Methodology, Investigation. **Xiaohu Luo:** Supervision, Resources. **Wei Liang:** Writing – review & editing, Supervision, Resources, Project administration, Funding acquisition, Conceptualization. **Lingling Jia:** Writing – review & editing, Writing – original draft, Supervision, Resources, Project administration, Methodology, Investigation, Funding acquisition, Conceptualization.

## Funding

This work was financially supported by funds from the 10.13039/501100001809National Natural Science Foundation of China (Grant No. 81901670), the Yongjiang Talent Introduction Programme (Young Talent sub-project, 2022A-159-G), the Key Project of Ningbo Science and Technology (2024Z184), the 10.13039/100007834Natural Science Foundation of Ningbo Municipality (2023J020; 2024J307), and the Development of *Clostridium butyricum* for Relieving Type 1 Diabetes (ZX2021000548).

## Declaration of competing interest

The authors declare that they have no known competing financial interests or personal relationships that could have appeared to influence the work reported in this paper.

## Data Availability

The raw Illumina sequence data of 16S rRNA are available in the sequence read archive (SRA) at NCBI under Bioproject accessions # PRJNA1410511. All data are available in the main text or the supplementary material.
