# Supplementary material for: Structural characterization of laminarin and its preventive effects on the gut microbiota–butyrate–pancreas axis in severe acute pancreatitis
Source: Food Chem X. 2026 Oct 30;40:104731. doi: 10.1016/j.fochx.2026.104731 (PMC13745741; doi:10.1016/j.fochx.2026.104731)
Supplement: Supplementary file 1 [file mmc1.docx]

*Supplementary Material*

**Captions**

**Table S1** HPAEC-PAD gradient elution conditions for monosaccharide separation.

**Table S2** The criteria of the pancreatic histological score.

**Table S3** The criteria of the colonic histological score.

**Table S4** Primer sequences used for RT-qPCR analysis.

**Fig. S1** PEG/PEO calibration curve for the molecular weight determination of laminarin.

**Fig. S2** Scanning electron microscopy (SEM) image of laminarin: Magnifications of 20,000× (A) and 50,000× (B).

**Fig. S3** Relative abundance of bacterial taxa at different taxonomic levels across groups: (A) *Firmicutes*; (B) *Proteobacteria*; (C) *Akkermansiaceae*; (D) *Lachnospiraceae*; (E) *Erysipelotrichaceae*; (F) *Desulfovibrionaceae*; (G) Enterorhabdus; and (H) Tyzzerella.

**Fig. S4** qPCR analysis of representative SCFA-producing bacterial taxa normalized to total bacterial: (A) *Faecalibaculum*, (B) *Ruminococcus*, (C) *Clostridium* cluster IV, and (D) *Clostridium* cluster XIVa.

**Fig. S5** Correlation between gut microbiota and systemic inflammation: (A,B) Spearman correlation between the top 20 most abundant bacterial genera and inflammatory cytokines (TNF-α, IL-6, IL-1β, CCL2) in pancreas (A), and colon (B). (C) Spearman correlation between the top 20 most abundant bacterial genera and inflammatory cytokines (TNF-α, IL-6, IL-1β) in the serum. (D) Spearman correlation between the top 20 most abundant bacterial genera and colonic tight junction proteins (Occludin, Claudin1, MUC2).

**Fig. S6** Concentrations of the other three SCFAs: (A) Isobutyric acid; (B) Valeric acid, and (C) Isovaleric acid.

**Table S1** HPAEC-PAD gradient elution conditions for monosaccharide separation.

| Time (min) | A (%) | B (%) | C (%) | Phase |
| --- | --- | --- | --- | --- |
| 0 | 95 | 5 | 0 | Initial equilibration |
| 26 | 85 | 5 | 10 | Linear gradient |
| 42 | 85 | 5 | 10 | Isocratic hold |
| 42.1 | 60 | 0 | 40 | Step change |
| 52 | 60 | 40 | 0 | Linear gradient |
| 52.1 | 95 | 5 | 0 | Step change |
| 60 | 95 | 5 | 0 | Hold |

**Table S2** The criteria of the pancreatic histological score.

|  | Score | Pathologic change |
| --- | --- | --- |
| Inflammation | 0 | 0-1 inflammatory cells |
|  | 1 | 2-10 inflammatory cells |
|  | 2 | 11-20 inflammatory cells |
|  | 3 | 20-30 inflammatory cells |
|  | 4 | > 30 inflammatory cells |
| Acinar edema | 0 | None |
|  | 1 | Focal expansion of interlobular septa |
|  | 2 | Diffuse expansion of interlobular septa |
|  | 3 | Acinar enlargement |
|  | 4 | Marked widening of interlobular septa |
| Vacuolization | 0 | None |
|  | 1 | < 20% acini with vacuoles |
|  | 2 | < 40% acini |
|  | 3 | < 60% acini |
|  | 4 | > 80% acini |
| Acinar necrosis | 0 | None |
|  | 1 | 1-4 necrotic cells |
|  | 2 | 5-10 necrotic cells |
|  | 3 | 11-16 necrotic cells |
|  | 4 | > 16 necrotic cells |

**Table S3** The criteria of the colonic histological score.

| Score | Inflammatory infiltrate | Goblet cell | Crypt density |
| --- | --- | --- | --- |
| 0 | None | Normal | Normal |
| 1 | Inflammatory cells increased | Decreased by < 10% | Decreased by < 10% |
| 2 | Infiltrates into submucosa | Decreased by 10-50% | Decreased by 10-50% |
| 3 | Infiltrates into transmural | Decreased by > 10% | Decreased by > 10% |

**Table S4** Primer sequences used for RT-qPCR analysis.

| Gene Name | Primer sequence | |
| --- | --- | --- |
|  | Forward | Reverse |
| β-Actin | CTACCTCATGAAGATCCTGACC | CACAGCTTCTCTTTGATGTCAC |
| TNF-α | AGGGTCTGGGCCATAGAACT | CCACCACGCTCTTCTGTCTAC |
| IL-1β | GCCACCTTTTGACAGTGATGAG | ATGTGCTGCTGCGAGATTTG |
| IL-6 | GAGGATACCACTCCCAACAGACC | AAGTGCATCATCGTTGTTCATACA |
| CCL2 | GCTGGAGAGCTACAAGAGGATCA | ACAGACCTCTCTCTTGAGCTTGGT |
| Occludin | ACGGACCCTGACCACTATGA | TCAGCAGCAGCCATGTACTC |
| MUC2 | GCTGACGAGTGGTTGGTGAATG | GATGAGGTGGCAGACAGGAGAC |
| Claudin-1 | GCTGGGTTTCATCCTGGCTTCTC | CCTGAGCGGTCACGATGTTGTC |
| Total bacterial | TCCTACGGGAGGCAGCAGT | GACTACCAGGGTATCTAATCCTGTT |
| *E. coli* | CATGCCGCGTGTATGAAGAA | CGGGTAACGTCAATGAGCAAA |
| *Ruminococcus* | CTTGCTGGACGATGACTGAC | CTCCGATTAAAGAGCGGTCAGA |
| *Faecalibacterium* | AGATGGCCTCGCGTCCGA | CCGAAGACCTTCTTCCTCC |
| *Clostridium* cluster IV | GCACAAGCAGTGGAGT | CTTCCTCCGTTTTGTCAA |
| *Clostridium* cluster XIVa | AAATGACGGTACCTGACTAA | CTTTGAGTTTCATTCTTGCGAA |
| butyrate kinase | CCATGCGTTAAACCAAAAAGC | AATACCTCCGCCCATATG |
| butyryl-  coenzyme A | GCIGAICATTTCACITGGAAYWSITGGCAYATG | CCTGCCTTTGCAATRTCI  ACRAANGC |

**Fig. S1**

**Fig. S1** PEG/PEO calibration curve for the molecular weight determination of laminarin.

**Fig. S2**

**Fig. S2** Scanning electron microscopy (SEM) image of laminarin: Magnifications of 20,000× (A) and 50,000× (B).

**Fig. S3**

**Fig. S3** Relative abundance of bacterial taxa at different taxonomic levels across groups: (A) *Firmicutes*; (B) *Proteobacteria*; (C) *Akkermansiaceae*; (D) *Lachnospiraceae*; (E) *Erysipelotrichaceae*; (F) *Desulfovibrionaceae*; (G) Enterorhabdus; and (H) Tyzzerella.

**Fig. S4**

**Fig. S4** qPCR analysis of representative SCFA-producing bacterial taxa normalized to total bacterial: (A) *Faecalibaculum*, (B) *Ruminococcus*, (C) *Clostridium* cluster IV, and (D) *Clostridium* cluster XIVa.

**Fig. S5**

**Fig. S5** Correlation between gut microbiota and systemic inflammation: (A,B) Spearman correlation between the top 20 most abundant bacterial genera and inflammatory cytokines (TNF-α, IL-6, IL-1β, CCL2) in pancreas (A), and colon (B). (C) Spearman correlation between the top 20 most abundant bacterial genera and inflammatory cytokines (TNF-α, IL-6, IL-1β) in the serum. (D) Spearman correlation between the top 20 most abundant bacterial genera and colonic tight junction proteins (Occludin, Claudin1, MUC2).

**Fig. S6**

**Fig. S6** Concentrations of the other three SCFAs: (A) Isobutyric acid; (B) Valeric acid, and (C) Isovaleric acid.
